# Investigating the evolution of the Arctic lineage of canine distemper virus circulating in Italy

**DOI:** 10.1186/s13567-026-01801-2

**Published:** 2026-07-04

**Authors:** Zsófia Lanszki, Francesco Pellegrini, Georgia Diakoudi, Violetta Iris Vasinioti, Gianvito Lanave, Maria Loredana Colaianni, Maria Stella Lucente, Canio Buonavoglia, Nicola Decaro, Ana Moreno, Enikő Fehér, Tamás Görföl, Gábor Kemenesi, Vito Martella

**Affiliations:** 1 National Laboratory of Virology, Szentágothai Research Centre, University of Pécs, Ifjúság útja 20., Pécs, 7624 Hungary; 2 Institute of Biology, Faculty of Sciences, University of Pécs, Ifjúság útja 6., Pécs, 7624 Hungary; 3 Department of Veterinary Medicine, University of Bari Aldo Moro, 70010 Valenzano, Italy; 4 ASL Bari-Local Health Authority Veterinary Service, Bari, Italy; 5 Istituto Zooprofilattico Sperimentale della Lombardia e dell’Emilia Romagna, Brescia, Italy; 6 Department of Microbiology and Infectious Diseases, University of Veterinary Medicine Budapest, Hungária krt. 23-25., Budapest, 1143 Hungary; 7 National Laboratory for Infectious Animal Diseases, Antimicrobial Resistance,, Veterinary Public Health and Food Chain Safety, István utca 2, Budapest, 1078 Hungary; 8 Department of Pharmacology and Toxicology, University of Veterinary Medicine, István utca 2, Budapest, 1078 Hungary

**Keywords:** *Paramyxoviridae*, *Morbillivirus*, *Morbillivirus canis*, inter-lineage recombination

## Abstract

Canine distemper virus (CDV) can cause fatal viral infection in domestic and wild animals globally. Several lineages are known, originating from distinct geographical regions and hosts, and can spread naturally or through human intervention into new geographic areas. The Arctic lineage was first described in carnivores of the Arctic ecosystems and subsequently reported in several European and Asian countries, yet its origin, evolution, and ecology remain partially unresolved. In this study, we generated genome sequence data of (*n* = 16) CDV strains of Arctic lineage collected from dogs in Italy over a nearly 15-year period, providing an extensive dataset to investigate the evolution of this particular lineage. We also generated genome data of seven Europe strains of another major lineage collected during the same period from red foxes (*n* = 3) and dogs (*n* = 4). Inter-lineage recombination events were identified in two CDV sequences. Sequence 2008 of the European lineage acquired a fragment from an Arctic lineage virus between the N and P genes. Sequence 2015 of the Arctic lineage displayed a more complex recombination pattern with fragments from Europe, America-2, and Rockborn lineages across multiple genes and hosts. Phylogenetic tree showed that the oldest Italian Arctic lineage from 2006 was more similar to the oldest Arctic CDV isolates, whilst a well-defined sub-cluster circulated from 2009 onwards in domestic and wild carnivores. These results provide novel insights into CDV evolution in Europe and emphasize the importance of ongoing genomic monitoring.

**Supplementary Information:**

The online version contains supplementary material available at 10.1186/s13567-026-01801-2.

## Introduction

Canine distemper virus (CDV) [virus species name: *Morbillivirus canis*] is a member of the genus *Morbillivirus* in the family *Paramyxoviridae*. The CDV genome is composed of a negative, single-stranded RNA genome, approximately 15690 nucleotides in length, and consists of six main genes [1]. At the 3ʹ end of the CDV genome, the N gene encodes the nucleocapsid (N) protein, which binds to the viral RNA to form the ribonucleoprotein (RNP) complex, protecting the RNA and enabling its replication and transcription. The P gene encodes the phosphoprotein (P), a cofactor for the viral RNA polymerase, and expresses accessory proteins (C and V) that help the virus evade the host immune system. Matrix (M) protein, the product of the M gene, facilitates viral assembly by linking the RNP to the viral envelope. The F gene encodes the fusion (F) protein, enabling the virus to fuse with host cell membranes for entry. The hemagglutinin (H) protein of the H gene allows the virus to attach to host cell receptors. Finally, the L gene at the 5ʹ end encodes the large (L) protein, the viral RNA-dependent RNA polymerase, responsible for genome replication and transcription [2, 3].

CDV is a highly contagious virus that can lead to severe illness with a high mortality rate in domestic dogs and wild animals. The virus is transmitted mainly by direct animal-to-animal contact through bodily fluids such as saliva, respiratory droplets, urine, and feces [1, 4]. CDV is known for its ability to infect a wide range of hosts in various mammals. Due to its broad host range and the ease of cross-species transmission, CDV has caused outbreaks and infections in wild and domestic animals worldwide [4–6]. This virus was detected in several mammalian species from different families, including Canidae, Felidae, Mustelidae, Procyonidae, and Ursidae in Europe [7]. CDV is a significant risk to the conservation of many carnivore species. The virus was identified as a contributing factor in the decline of several wild mammalian species, chiefly those that are already facing multiple challenges [8, 9]. For example, CDV has been found to pose a significant threat to the conservation of Apennine wolves (*Canis lupus*) and Brown Bear (*Ursus arctos marsicanus*) in Italy [10, 11].

CDV is a monotypic virus as defined by polyclonal antisera, and a single exposure to the virus confers long-term immunity. CDV uses the H protein for attachment to receptors on the cell in the first step of infection and an adequate host immune response against the H protein may prevent CDV infection [12, 13]. Comparative studies of CDV strains have revealed that the H gene is subjected to higher genetic/antigenic variation than other CDV genes and that sequence variation may affect neutralization-related sites with disruption of important epitopes [14–18].

The high genetic diversity of the H gene enables the distinction of CDV field strains into different genetic lineages and contributes to gaining insights into the epidemiology and evolution of the virus [6, 19]. These strains of variable CDV lineages can naturally spread into new geographical regions, such as through wild-animal movements between countries or through human-related activities like the animal trade. The geographical dissemination of viruses belonging to different CDV lineages is a significant concern.

One example is the Arctic lineage that has spread throughout Europe over the past two decades. The initial description of Arctic lineage dates back to the late 1980 s, when morbillivirus-like epizootics were observed in seals in Northern Europe and Siberia [20–22]. Subsequent genetic analysis demonstrated that the morbillivirus epizootics were epidemiologically distinct, as they were caused by a phocine distemper virus, PDV-1, and by a CDV-like strain, PDV-2, respectively. Also, PDV-2-like strains continued to circulate in Baikal seals at least until 1992. Feral and domestic dogs around Lake Baikal were suspected to be the source of infection for freshwater seals [23]. Around the same years, 1988, a CDV strain, GR88, was detected from a virgin soil outbreak in a sledge dog population in a remote Inuit settlement in Arctic Northern Greenland [15]. Strain GR88 was subsequently found to be closely related to strain PDV-2. Accordingly, both GR88 and PDV-2 were regarded as distinct CDV lineages circulating across the Arctic ecosystem in susceptible species, such as polar bears and arctic foxes [16, 24–26]. Subsequent studies have revealed that CDVs of this lineage are common in European domestic and wildlife mammals susceptible to CDV [27–29]. However, the epidemiological and molecular data on Arctic CDV are still scattered, limiting our understanding of the evolution of this lineage. Complete genome analysis is believed to be much more effective for uncovering genomic-level evolutionary patterns, specific mutations, and recombination events than investigations based on a single gene target (i.e. the H gene).

Recombination plays a pivotal role in driving the evolutionary dynamics of CDV, significantly contributing to its genetic diversity and adaptive evolution. Recombination events have been documented not only among wild-type, but also between field and vaccine strains, resulting in the emergence of recombinant variants [30]. Since the H gene is the most variable region in the CDV genome, making it a primary target for molecular typing and phylogenetic classification of CDV lineages [14], recombination has been investigated preferably in this region [31, 32].

Next-generation sequencing (NGS) technologies have become a useful tool for the detection and characterization of pathogens in domestic and wild animals [33–35]. Genomic studies can help to better understand the geographic and interspecies spread of viruses, and are not only suitable for the current epidemics, but also for the investigation of disease outbreaks in the past [36].

In this study, we aimed to investigate the history of the Arctic CDV lineage in Italy between 2005 and 2019 through complete genome sequencing-based retrospective analysis of a collection of archival CDV strains. Furthermore, a comprehensive recombination analysis was performed to detect potential genetic crossover events within the viral genome, thus, to gain insights into the evolutionary mechanisms of CDV strains from Arctic lineages.

## Materials and methods

### Sample collection and nucleic acid extraction

Twenty-three samples from dogs (*Canis lupus familiaris*) and red foxes (*Vulpes vulpes*) were collected between 2005 and 2019 in Italy (Additional file 1). In order to maximize the viral RNA extraction yield, tissue samples were homogenized (50% w/v) in Dulbecco’s Modified Eagle Medium (D-MEM, Corning, NY, USA) using a TissueLyser (Qiagen, Hilden, Germany) at 25 Hz for 3 min. The homogenates were centrifuged at 20000 rcf for 3 min. Nucleic acids were extracted from 400 µL of the supernatant of homogenized tissue samples (lung, spleen, lymph nodes, or brain) using the GeneJET Viral DNA/RNA Purification Kit (Thermo Fisher Scientific, Waltham, MA, USA), according to the manufacturer’s instructions. The DNA and RNA obtained were eluted in 50 µL of buffer and stored at −80 °C until further use.

### CDV screening by RT-qPCR

An RT-qPCR targeting the phosphoprotein (P) gene was used to screen for CDV, as previously described [37]. Reactions were carried out using the One-Step RT-qPCR System with Platinum Taq DNA Polymerase (Invitrogen, Carlsbad, CA, USA) on a CFX96 Touch Real-Time PCR Detection System (Bio-Rad Laboratories, Hercules, CA, USA). Samples with a cycle threshold (Ct) value below 35 were selected for complete genome sequencing.

### Nanopore sequencing and data analysis

The complete genome sequencing was performed with MinION Nanopore sequencing technology (Oxford Nanopore Technologies, UK). We used a previously published universal pan-genotype CDV-specific amplicon-based sequencing method designed for CDV [36]. The detailed protocol and the primers are available at our laboratory’s protocols.io page [38]. To generate overlapping genome fragments, two sets of primers were used to produce different lengths of amplicons (1000 bp and 2000 bp). The sequencing runs were performed on a R9.4.1. (FLO-MIN106D) flow cell with the AMX-F motor protein from SQK-LSK110 kit (Oxford Nanopore Technologies, UK) on MinION Mk1B device.

Base-calling and demultiplexing of the raw data was performed with Guppy software (version 6.0.1.) using the super accurate base-calling model and default parameters with the “barcode_both_end” option. The generated reads were further processed, as 50 bases pairs were trimmed from both ends, and the dataset was filtered to eliminate the short and chimeric sequence reads. All generated reads from a sample were mapped to the reference sequence GenBank accession number MN267060 using Geneious Prime (version 1.6.0., Biomatters, New Zealand). Finally, the preconsensus sequences were polished using Medaka (version 2022.1.1) to generate the final consensus sequences, with a minimum coverage of 100 × per nucleotide position to ensure accuracy.

### Sequence analysis

A set of 61 full-length amino acid sequences of the hemagglutinin of Arctic CDVs was aligned using Clustal Omega 1.2.3 [39]. The potential glycosylation sites (N-X-T/S) and the receptor binding residues were mapped and visually inspected.

### Phylogenetic analysis

To perform the phylogenetic analysis, the related sequences were collected from the NCBI database and assembled with the novel sequences in this study (Additional file 1). Complete genome and H gene sequences were aligned with MAFFT v.7.505 [40]. Model parameters (complete genome: GTR + I + G; H gene: TIM1 + G4m) were determined with ModelTest-NG v.0.1.7 [41]. The tree was generated using RaxML-NG v.1.2.0 with 1000 bootstraps [42]. The resultant tree was edited in iTOL [43].

### Recombination analysis

The dataset for recombination analysis was composed with the novel sequencing data of this study and from already existing 255 complete and nearly complete genomic data from NCBI database. Recombination was investigated using the Recombination Detection Program 4 (RDP4; based on the RDP, GENECONV, BootScan, MaxChi, Chimaera, SiScan, 3Seq methods) [44]. Recombination events supported by at least three detection methods and phylogenetic trees generated within RDP4 were considered. A Genetic Algorithm for Recombination Detection was applied to evaluate recombination breakpoints [45].

## Results

### Sequencing of CDV positive samples

A total of twenty-three CDV positive samples collected between 2005 and 2019 from domestic dogs and red foxes in Italy were included in this study. The samples represented different years and hosts, allowing the investigation of temporal and interspecies distribution patterns of the circulating CDV lineages. Complete genomes were obtained from all samples and subsequently deposited in the GenBank with accession numbers PX734733-PX734755. The distribution of the samples by year, host species, and the lineage of the CDVs established through genome sequencing is represented in Table 1.

**Table 1:** Summary data on CDV-positive samples

Sample ID	Year	Host species	Lineage	RT-PCR Ct value	Number of reads	Mean coverage	Accession number
199/05	2005	dog	Europe	16.99	266,077	15,115.4	PX734736
224/05	2005	dog	Europe	22.01	180,671	13,284.8	PX734739
152/06	2006	dog	Arctic	19.98	413,719	61,737.4	PX734734
364/06	2006	dog	Europe	23.09	16,274	1,011.6	PX734747
290/08	2008	dog	Europe	24.67	153,380	8,941.4	PX734742
350/09	2009	red fox	Europe	22.24	259,393	20,098.2	PX734745
403/09	2009	red fox	Europe	21.77	263,042	35,138.3	PX734748
417/09	2009	dog	Arctic	32.01	45,151	2,243.6	PX734750
145/10	2010	red fox	Europe	25.22	333,164	23,441.4	PX734733
202/10	2010	dog	Arctic	25.62	190,416	13,527.3	PX734738
97/11	2011	dog	Arctic	27.00	119,397	7,709.1	PX734754
201/13	2013	dog	Arctic	22.45	280,244	21,602.3	PX734737
338/13	2013	dog	Arctic	26.55	256,22	18,195.0	PX734744
359/13	2013	dog	Arctic	22.94	384,985	35,461.5	PX734746
259/14	2014	dog	Arctic	17.71	11,233	791.5	PX734741
24/15	2015	dog	Arctic	16.54	481,428	52,335.3	PX734740
310/15	2015	dog	Arctic	30.59	2,220	113.0	PX734743
99/16	2016	dog	Arctic	30.04	8,898	382.0	PX734755
190/16	2016	dog	Arctic	19.12	299,719	35,083.0	PX734735
856/17	2017	dog	Arctic	28.86	8,595	402.3	PX734753
592/18	2018	dog	Arctic	23.28	141,600	10,989.0	PX734752
412/19	2019	dog	Arctic	21.69	209,853	15,920.6	PX734749
425/19	2019	dog	Arctic	23.63	25,904	3,047.3	PX734751

### Phylogenetic and sequence analysis

**Figure 1:**
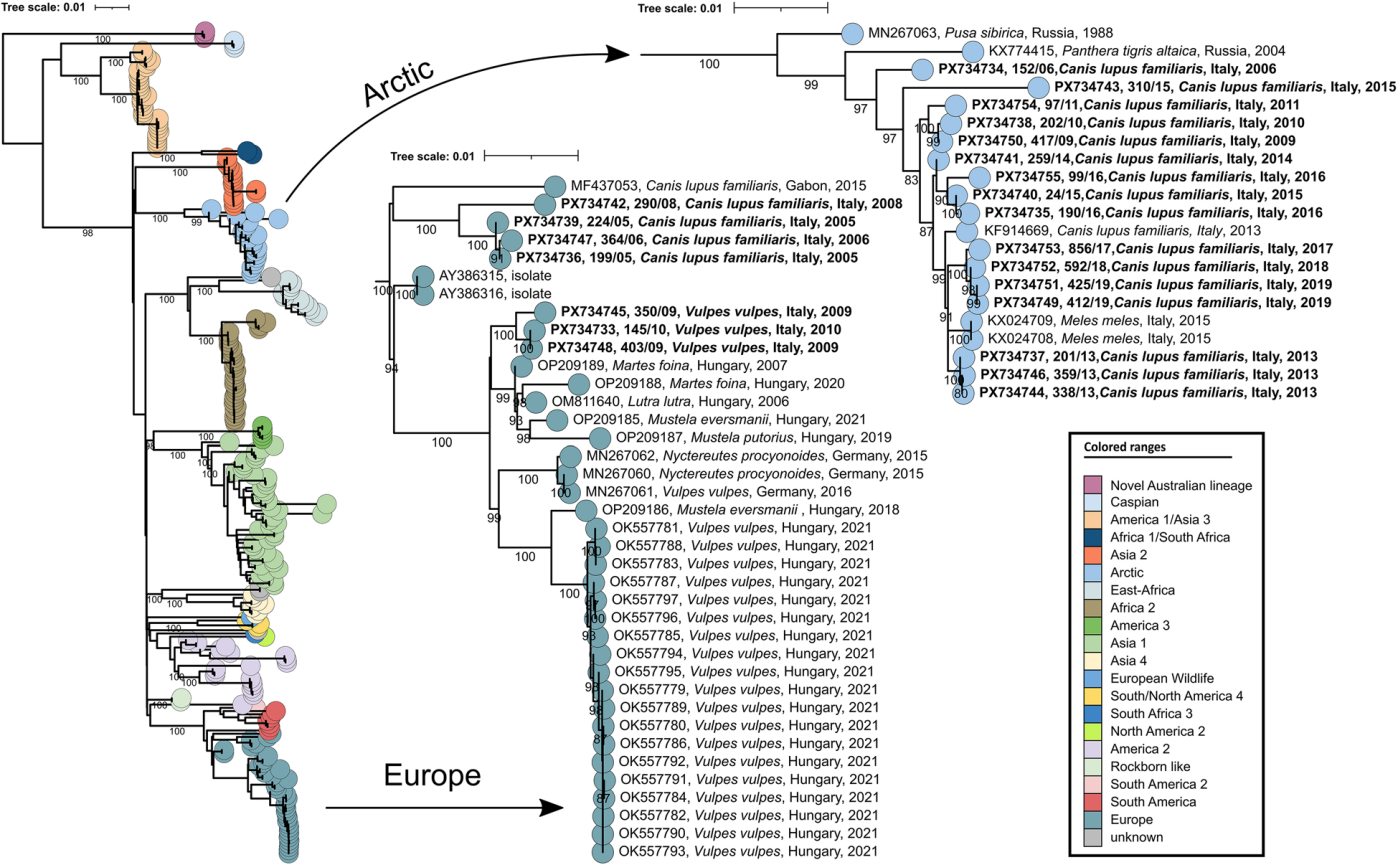
**Maximum likelihood phylogenetic tree based on 255 CDV complete genomes.** Phocine distemper virus (PDV) (GenBank accession number: KY629928) was used as an outgroup to root the phylogenetic tree. Arrows indicate expanded portion of Arctic and Europe lineages. The novel Italian sequences are shown in bold.

Phylogenetic analysis was performed on the novel sequence data from Italy to assess genomic clustering of these strains. Based on the complete genome phylogenetic analysis, 7/23 and 16/23 sequences belonged to the Europe and Arctic lineage, respectively (Figure 1). Regarding the dog origin sequences, 4 clustered to the Europe and 16 to the Arctic lineage. The 3 red fox origin sequences were all grouped into the Europe lineage, which clustered more distantly compared to the CDV genomes identified in dogs.

**Figure 2:**
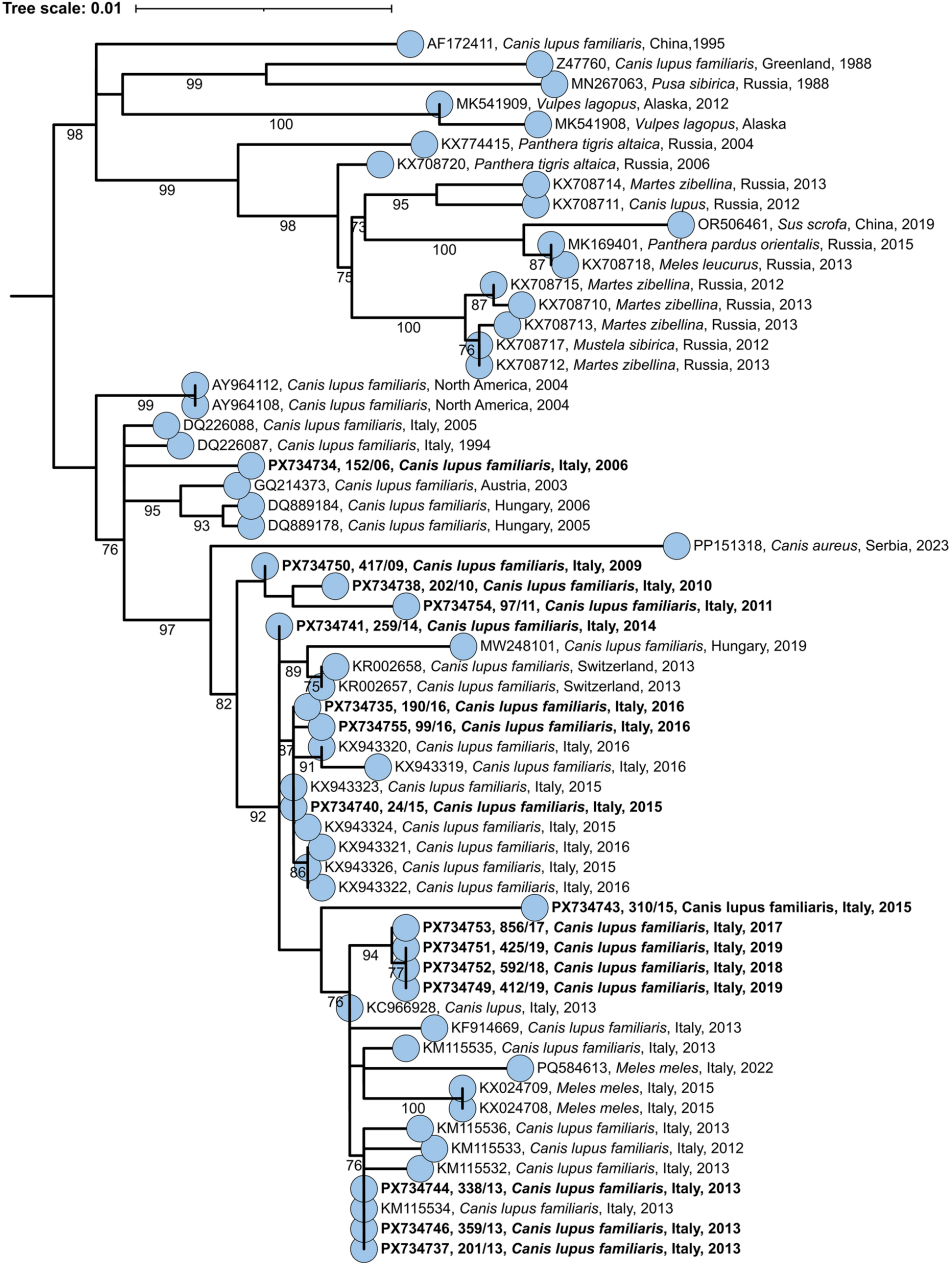
**Maximum likelihood phylogenetic tree based on 61 H gene sequences of Arctic CDVs.** The novel Italian sequences are shown in bold.

Since the H gene is the most common target for CDV sequencing/characterization, a phylogenetic tree (Figure 2) was also generated using a larger data set (*n* = 61) of Arctic CDV strains. In this analysis, the oldest Italian Arctic strain (152/06) sequenced in this study was more similar to the oldest Arctic CDV isolates, whilst a well-defined sub-cluster circulated from 2009 onwards in domestic and wild carnivores.

On sequence analysis of the inferred amino acid sequence of the H protein sequence, the glycosylation sites (Asn149, Asn309, Asn391, Asn422, Asn456, and Asn587 and Asn603) were highly conserved across Arctic strains, with the exception of a cluster of CDVs identified in mustelids in Russia 2012–2013 in which the site Asn309 on the head domain was disrupted [46]. An additional glycosylation site (Asn298) was a signature of two CDV strains detected in Alaska 2012–2014 in foxes (accessions MK541908 and MK541909). The receptor binding sites (SLAM and nectin-4) were highly conserved. Interestingly, in all European Arctic CDVs, there was a change, 538-Ile to Val, in the hydrophobic pocket on the side of the H-protein's globular head, between the critical residues Tyr537 and Tyr539, that interact with the V-domain of nectin-4.

### Recombination analysis

Using the global dataset of complete or near-complete genomes, several inter-lineage recombination events were identified. Fifteen cases were well supported statistically and by the phylogeny and were examined in more detail to understand the frequency and nature of inter-lineage recombination on a global scale (Table 2).

**Table 2:** **The table represents four statistically well-supported recombination events calculated using a dataset of 255 nearly complete canine distemper virus sequences**

	Recombinant	Major parent	Minor parent	Affected region	No. of stat. methods, p val
1	***310/15*** ***dog, (Arctic)***	***259/14*** ***dog, (Arctic)***	***350/09*** ***red fox (Europe)***	3’P-M-5’F (nt 2930–4937)	7, 10^–10^–10^–42^
2	***310/15*** ***dog, (Arctic)***	KF914669 (Arctic) dog, Italy, 2013	MT448054 (Rockborn) masked civet, China, 2019	3’F-5’H (nt 6700–7372)	7, 10^–6^–10^–16^
3	***310/15*** ***dog, (Arctic)***	***359/13*** ***dog, (Arctic)***	AF164967 (America-2) dog, USA	N-P (nt 785–2886)	7, 10^–2^–10^–14^
**4**	***290/08*** ***dog, (Europe)***	***199/05*** ***dog, (Europe)***	***425/19*** ***dog, (Arctic)***	3’N-3’P (nt 1231–2884)	7,10^–6^–10^–32^

The novel strains are written in bold and italic.

*N* nucleoprotein, *P* phosphoprotein, *M* matrix protein, *F* fusion protein, *H* hemagglutinin, *L* large polymerase protein, *IGR* intergenic region.

**Figure 3:**
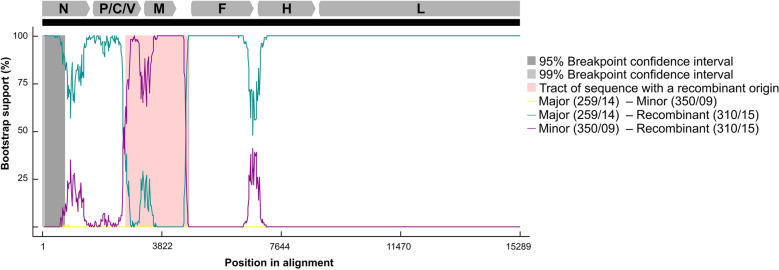
**Recombination event 1 represented by the Recombination Detection Program 4 software.**

**Figure 4:**
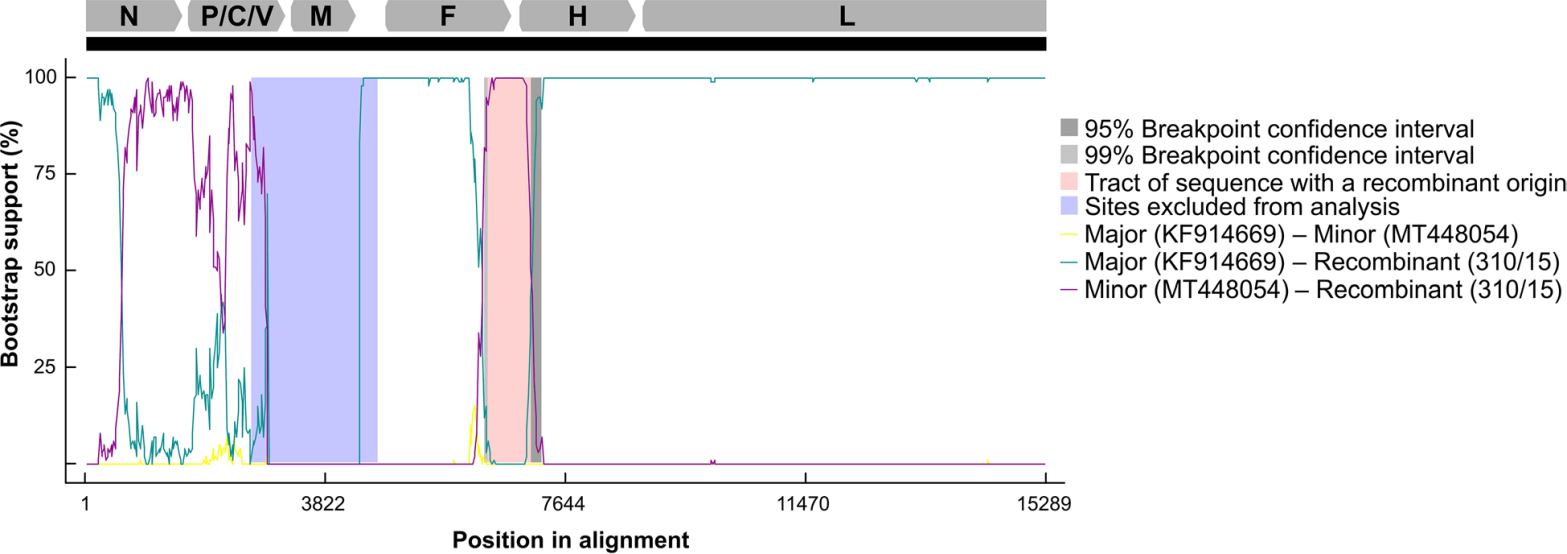
**Recombination event 2 represented by the Recombination Detection Program 4 software.**

**Figure 5:**
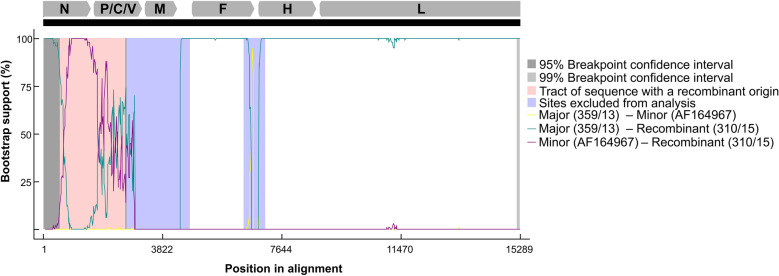
**Recombination event 3 represented by the Recombination Detection Program 4 software.**

**Figure 6:**
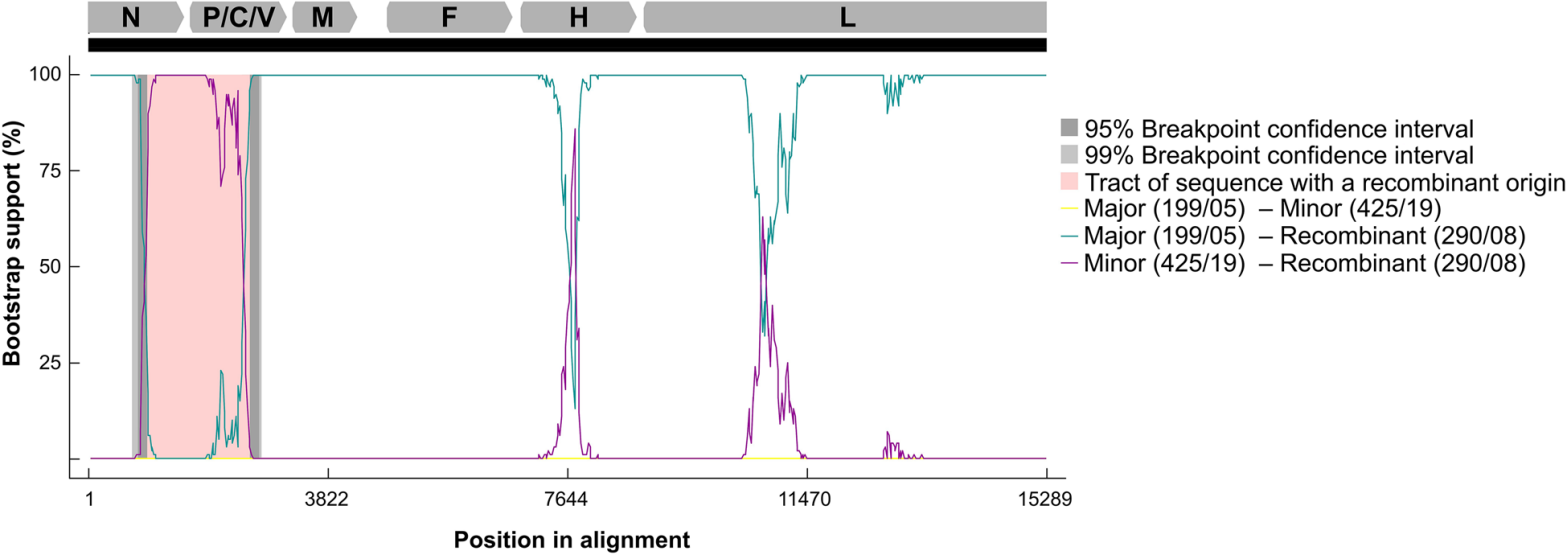
**Recombination event 4 represented by the Recombination Detection Program 4 software.**

Four events involved here characterized strains as recombinants (strain 310/15: event 1, 2 and 3; strain 290/08: event 4) (Figures 3, 4, 5, 6). CDV strain 350/15 exhibited three recombinant regions. The major parents were three different Arctic strains, two of which were described in the present study (strain 259/14: event 1; strain 359/13: event 3). The minor parent in event 1 was a new strain originating from red fox and belonging to the Europe lineage (strain 350/09) that could provide the complete M region. The minor parents listed in event 2 and 3 belonged to other lineages, namely the Rockborn and the America-2. The recombinant regions overlapped the F/H IGR with breakpoints in the 3’F and 5’H for the event 2, while the breakpoints were located in the N-P for the event 3. The recombinant of the event 4 clustered with strains of Europe lineage, and both the minor and major parents were novel strains, the minor grouping with the Arctic lineage.

## Discussion

The application of molecular sequencing and phylogenetic analysis to the H gene has revolutionized our understanding of CDV. It has become clear that CDV is not a single, antigenically stable entity. Instead, it is a dynamic collection of distinct viral populations, or lineages, that often exhibit strong geographical clustering. These lineages are named based on the continent or region of their initial or predominant detection [47].

The Arctic lineage was first identified in 1988 from a mass mortality of Baikal seals (*Pusa sibirica*) in Lake Baikal, Russia [23]. In the same year, a genetically related strain, GR88, was isolated from a population of sledge dogs in Northern Greenland [15]. The discovery of these related viruses in both marine mammals and domestic canids across the vast Arctic expanse pointed to a deep-rooted presence in this ecosystem. Indeed, a study of Arctic foxes (*Vulpes lagopus*) on the North Slope of Alaska between 2012 and 2013 found that CDV was a cause of mortality. Phylogenetic analysis of the H gene from foxes revealed that the virus belongs to the Arctic lineage [48], confirming that the lineage is enzootic, or persistently maintained, within wildlife populations of the Arctic.

In dogs, the Arctic lineage was first reported in samples collected in 2004 and 2005 published in a 2006 Italian study [28]. On sequencing of the H gene, two viruses were highly related to strain GR88. Retrospective molecular studies suggested that the lineage was largely circulating in the domestic dog population in the early 2000s. On screening of 36 dogs, sampled between 2002 and 2006, 14/36 (38.9%) were genotyped as Arctic and 22/36 (61.1%) as Europe lineage [49]. Subsequent studies have consistently confirmed the long-term persistence and co-circulation of Arctic strains in Italy across both domestic and wildlife hosts, supporting the notion that this lineage has become established within the local epidemiological landscape.

In the winter of 2013, a severe epizootic outbreak occurred in the protected Apennine wolf (*Canis lupus*) population in central Italy. This event marked the first confirmed outbreak of the Arctic lineage in a European wild population and resulted in the deaths of at least 20 wolves. The source of the outbreak was traced back to domestic dogs. The viral sequences from the wolves were found to be nearly identical to those circulating in local domestic dogs, and the epizootic in wolves occurred concurrently with numerous reports of clinical distemper in unvaccinated shepherd, feral, and pet dogs in the same region. The virus was also identified in badgers (*Meles meles*) in the same Apennine areas in the fall of 2015 [50]. This provided unequivocal evidence of a spillover event from a domestic reservoir to vulnerable wildlife. The sustained circulation of Arctic lineage viruses in this multi-host system suggests that ecological connectivity between domestic and wild carnivore populations, together with potential host adaptation and immune-mediated selection pressures, may have contributed to the successful stabilization of this lineage in Italy.

Since then, Arctic CDVs have been reported in several countries of the Northern hemisphere in domestic and wildlife animals in observational and retrospective studies. The Arctic lineage was reported in dogs in Hungary in 2005–2006 [51], Austria in 2003 [52], Switzerland in 2013 [53], Turkey in 2018 [54], and Iran in 2011 [55]. Also, it was described in jackals (*Canis aureus*) in Serbia, 2019–2023 [56]. In the USA, the Arctic lineage was described in dogs, between 2004 and 2007, in Illinois [57] and Missouri [58, 59]. The virus was also reported in Amur tigers (*Panthera tigris altaica*) in Sichotė-Alin' mountains, far Eastern Russia, in the years 2001–2010 [60], in a wild far eastern leopard (*Panthera pardus orientalis*) in 2015 in the Russian territory of Primorskii Krai [61], and in red foxes (*Vulpes vulpes*) in China in 2005 [62].

In this study, we generated genome sequence data for n = 16 Arctic lineage CDV strains collected from dogs in Italy over a nearly 15-year period, providing a unique observatory to gather insights into the evolution of this enigmatic CDV lineage. Also, we generated genome data of n = 7 CDV strains of another major CDV lineage (Europe), collected in the same period from red foxes (*n* = 3) and dogs (*n* = 4). Mapping the regional origin of the novel Italian strains (Additional file 1) confirms distinct spatial clustering. Within the European lineage, sequences from dogs (2005–2006) and foxes (2009–2010) segregated into different geographical territories of southern and northern Italy, respectively. Similarly, Italian arctic lineage strains from 2013 outbreaks clustered together, apart from the 2017–2019 canine outbreaks, underscoring the persistence and sub-cluster evolution in the south. Inter-lineage recombination events were identified in two CDV strains. Strain 290/08, of Europe lineage, had a nearly 1600 nt-long portion acquired by recombination with an Arctic strain, encompassing the N/P IGR. Strain 310/15, Arctic lineage, had a unique, more intricate recombination pattern, with large genome fragments derived from lineage Europe (affecting P and F genes), lineage America-2 (affecting the N and P genes), and Rockborn (in the F/H IGR). According to our available clinical records, the dog from which the recombinant strain 310/15 was isolated had a history of previous vaccination against CDV, but no vaccine had been administered within the 12 months prior to the onset of the disease. Consequently, the animal was infected by an already circulating field recombinant strain, which eluded vaccine‑induced immunity. These recombination events may have consequences, potentially affecting viral properties such as virulence, host tropism, and immune evasion [3, 63]. Exchanges in either structural or antigenic genes could alter receptor binding or antigenicity, influencing viral fitness, transmission dynamics, and interactions with host immune responses [64].

Previous publications have proposed recombination as a force in the evolution of the CDV [32, 62, 65, 66]. Since CDV H gene was considered as a basis for strain classification, this gene was the main target of recombination analysis. Intragenic exchange has been found in this region among strains of variable lineages and hosts [62, 63]. Another example was the recombination between dog-origin CDV strains of the Asia-1 and America-2 lineages, resulting in the emergence of an Asia-1 lineage strain recombinant in the H gene [66]. Traces of intragenic recombination have been detected within the F region of CDV strains collected from dogs, in which the recombinant and major parent strains belonged to the Arctic lineage, while the minor parent belonged to the European wildlife lineage [32]. However, recombination analysis of long genomic regions, also performed in this study, revealed exchanges spanning more genomic regions, thus highlighting the importance of whole genome-based sequence investigations [30, 65–69].

Of interest, the genome of the recombinant strain 310/15 embedded a genomic element derived from CDV America-2 lineage, common in the North American continent but never described in Europe. Also, it contained a genome element, a portion of the H gene, derived from a Rockborn-like CDV [70]. Strain Rockborn was isolated in the 1950s from a dog, after large epidemics caused by CDV in Sweden in both vaccinated and unvaccinated dogs [71]. The virus was attenuated after serial passages in tissue cells [72] and distributed as a vaccine globally after 1962. In the 1970 s and early 1980 s, post-vaccinal encephalitis, related to the Rockborn vaccine strain, was reported in dogs from various parts of Britain [73, 74]. Suspected cases of post-vaccinal encephalitis were also reported in 1995 in the USA in dogs vaccinated with a polyvalent vaccine containing the strain Rockborn. Consequently, the Rockborn strain was withdrawn from several markets after the mid-1990s [75]. However, Rockborn-like CDV is still present in some vaccines, and Rockborn-like viruses may be sporadically recovered from dogs or other carnivores with distemper disease, suggesting either residual virulence of some vaccine batches or the circulation of analogous viruses in hidden ecological niches [76]. Crucially, the Rockborn strain still retains enhanced replicative fitness and prolonged viral persistence in host tissues [76]. Over time, the co-circulation with endemic wild-type strains, such as those of the Arctic lineage, provides the ideal biological setting for homologous recombination [69]. This could account for the origin of the parental Rockborn-like strain, donor of the 5’end of the H gene in strain 310/15. These findings highlight the importance of continuous genomic surveillance of both field and vaccine-associated strains, as recombination involving vaccine-like viruses may have implications for vaccine strain monitoring and the long-term effectiveness of immunization strategies. Although this recombinant CDV strain was not detected in Italy in subsequent years, this underscores the potential for the emergence of novel variants.

We also generated a tree based on the H gene (Figure 2), including all the Arctic CDV strains in the GenBank repository. In this analysis, two major clusters were defined, with an apparent geographical pattern. One cluster included only CDV strains from Greenland, Alaska, Russia, and China. The other cluster comprised mostly European strains and some North American viruses. Within this cluster, a well-defined sub-cluster circulated among domestic and wild carnivores from 2009 onwards, suggesting the introduction or selection of CDV variants. Direct inspection of the H protein sequence did not show peculiar variations in the glycan shield since the glycosylation sites (Asn149, Asn309, Asn391, Asn422, Asn456, and Asn587 and Asn603) were highly conserved across Arctic strains, with the exception of a cluster of CDVs identified in mustelids in Russia 2012–2013 in which the site Asn309 was disrupted [46]. The glycan shield is a critical structural feature of morbilliviruses, including CDV, consisting of N-linked oligosaccharides that coat the surface of the H and F glycoproteins [77]. This shield serves three primary biological functions, ensuring structural integrity, modulating receptor interactions, and facilitating immune evasion. Also, the receptor binding sites (SLAM and nectin-4) were highly conserved. Interestingly, in all European Arctic CDVs, there was a change, 538-Ile to Val, in the hydrophobic pocket on the side of the H-protein's globular head, between the critical residues Tyr537 and Tyr539, that interact with the V-domain of nectin-4 [78]. In the biology of CDV, Nectin-4 functions as the essential epithelial receptor, determining the virus's tissue tropism, systemic spread, and shedding [79]. While the virus initially enters the host via immune cells using the SLAM (CD150) receptor, nectin-4 is required for the subsequent invasion of epithelial tissues [80]. This dual-receptor mechanism represents the hallmark of morbillivirus pathogenesis. In dogs, nectin-4 is also expressed in specific neuronal populations and likely is involved in the development of neurological distemper and encephalitis. The molecular fit between the H-protein and nectin-4 is also a primary determinant of the species barrier [3]. In conclusion, this study provides novel genome-scale insights into the evolution of Arctic and European CDV lineages circulating in domestic and wild animals in Europe. By generating and analysing complete genome sequences, we demonstrate that recombination is not limited to short regions of the H gene but can involve extensive genomic fragments originating from multiple CDV lineages. These findings highlight the complex evolutionary dynamics shaping CDV diversity and emphasize the added value of complete genome sequencing for resolving viral ancestry, detecting hidden recombination events, and improving molecular surveillance. Future studies incorporating phylogeographic approaches could provide deeper insight into the geographic origin and spatial spread of CDV strains identified in Italy. Integrating genome-wide analyses into routine monitoring efforts will be essential to better understand CDV transmission, evolution, and its implications for both domestic animal health and wildlife conservation.

## Supplementary Information

**Additional file 1 Metadata associated with 255 CDV sequences retrieved from NCBI, used for complete genome phylogenetic and recombination analyses.**

## Data Availability

The data presented in this study are openly available. Sequence data are deposited under NCBI GenBank accession numbers PX734733-PX734755. All data analyzed during this study are included in the results section.
